# Aurora Image Classification with Deep Metric Learning

**DOI:** 10.3390/s22176666

**Published:** 2022-09-03

**Authors:** Takeru Endo, Mitsuharu Matsumoto

**Affiliations:** Department of Informatics, The University of Electro-Communications, 1-5-1, Chofugaoka, Chofu-shi, Tokyo 182-8585, Japan

**Keywords:** aurora, deep learning, image classification, metric learning, machine learning

## Abstract

In recent years, neural networks have been increasingly used for classifying aurora images. In particular, convolutional neural networks have been actively studied. However, there are not many studies on the application of deep learning techniques that take into account the characteristics of aurora images. Therefore, in this study, we propose the use of deep metric learning as a suitable method for aurora image classification. Deep metric learning is one of the deep learning techniques. It was developed to distinguish human faces. Identifying human faces is a more difficult task than standard classification tasks because this task is characterized by a small number of sample images for each class and poor feature variation between classes. We thought that the face identification task is similar to aurora image classification in that the number of labeled images is relatively small and the feature differences between classes are small. Therefore, we studied the application of deep metric learning to aurora image classification. As a result, our experiments showed that deep metric learning improves the accuracy of aurora image classification by nearly 10% compared to previous studies.

## 1. Introduction

Auroras are an atmospheric luminous phenomenon that occurs when solar winds collide with the Earth’s magnetosphere. Observations of auroras can therefore provide insight into magnetospheric processes. In order to observe the state of the magnetosphere, many scholars have explored the shapes and characteristics of auroras. In recent years, many attempts have been made to automatically determine the shape of auroras.

All-sky imaging equipment is used to observe auroras. The amount of data collected is enormous, reaching up to 20 TB per year [1]. In order to analyze these aurora images, each image needs to be assigned annotations. Many researchers have annotated these images and created datasets. In 2012, Yang et al. analyzed these aurora images using a hidden Markov model [2]. Recently, it has become common to assign classes to aurora images by supervised learning using neural networks. Kvammen et al. [3] created a highly accurate classifier by supervised learning using convolutional neural networks. They achieved a 0.90 F1 score on the aurora image classifications using the residual network (ResNet) [4].

One of the characteristics of aurora images is that there is little change in shape between the classes. Unlike typical image classifications, aurora images are difficult to classify. Furthermore, the difficulty of labeling images makes it difficult to prepare large amounts of training data. We considered these two difficulties in aurora image classification to be common to the face identification task. Therefore, we attempted to apply deep metric learning [5], which is widely used in face identification, to aurora image classification.

Schroff et al. [5] showed that deep metric learning achieves high accuracy in face identification. They defined a new type of loss function called triplet loss (Triplet Margin Loss). Metric learning methods intentionally increase the distance between inter-class samples and decrease the distance between intra-class samples. This allows the DNN to generate discriminative features.

To evaluate the effectiveness of deep metric learning for the classification of aurora images, we conducted experiments on a dataset with six class labels. The experimental procedure is as follows.

1. We trained a convolutional neural network using deep metric learning.
2. We encoded images into feature vectors using the pre-trained CNN.
3. We classified the feature vectors obtained in 2. We encoded images into feature vectors using the pre-trained CNN. In this study, we used the ridge regression classifier and the classification using Mahalanobis distance.

To test the usefulness of our proposed method, we conducted two comparative experiments. One is a previous study using inceptionV4 and ridge regression [6]. The other is a method that uses a convolutional neural network [3]. From the experimental results, we confirmed that our method using deep metric learning improves accuracy by nearly 10% compared to previous studies. From these results, we can conclude that, based on the similarities between the face identification task and the aurora image classification, we were able to verify our hypothesis that metric learning works well for the aurora image classification.

## 2. Related Works

### 2.1. Aurora Image Classification with Deep Learning

Syrjäsuo et al. [7] were the first to introduce computer vision technology to classify aurora images, classifying them into the arc, patchy aurora, omega band, and north–south structure according to their shape information. Subsequently, they studied the extraction of auroral image features by Fourier transform [8]. However, these methods could not handle all aurora images in a general way. To solve this problem, Han et al. [9] used Scale Invariant Feature Transform (SIFT) to classify aurora images. In 2014, Bing et al. [10] combined the Latent Dirichlet Allocation (LDA) model with saliency information to present an aurora image classification model based on a topic model. However, feature extractors and classifiers in these classification models were separated. With the development of deep learning, it has become possible to perform automatic feature extraction and image classification in a single step. In particular, recent years have shown the usefulness of convolutional neural networks (CNN) for classifying aurora images [6,11,12]. In 2015, Clausen et al. [6] used Inception-v4 for aurora image classification. They used the THEMIS Ground-Based All-Sky Imager (ASI) dataset, which is a dataset with labels for six classes: Arc, Diffuse, Discrete, Cloudy, Moon, and Clear. They showed that classification using convolutional neural networks is effective for aurora image identification. Then, Zhong et al. [13] used a dataset with four discrete labels (Arc, Drapery, Radial, and Hotspot) taken by the Yellow River Station (YRS) to classify aurora images. They used three CNN models, AlexNet [14], VGG16 [15], and ResNet18 [4]. As a result, they achieved an 87.8% accuracy rate with ResNet18. However, a classifier that takes into account more detailed features of auroral images was needed to classify auroral shapes more accurately. Therefore, Niu et al. [16] proposed a classification method using semantic segmentation. Furthermore, in 2020, Yang et al. [17] proposed a classification method using Spatial Transformer Networks (STN) and large-margin-softmax loss. They used 8000 images from the YRS aurora images for training and 2184 images for test data. They succeeded in classifying auroral images with a 93.7% accuracy rate. In addition, Uchino and Matsumoto [18] showed that data augmentation using GAN, a generative model, can improve image classification accuracy. Furthermore, in 2021, Sado et al. [19] researched the effectiveness of transfer learning of CNNs pre-trained on ImageNet for the Oslo Auroral THEMIS (OATH) dataset of all-sky images. They demonstrated the usefulness of the transfer learning of the pre-trained CNN model. In 2022, Qiuju et al. [20] reported that CNNs can be used to accurately classify aurora images, even with small amounts of labeled data. They also used AlexNet, VGG, and ResNet for CNNs.

Deep learning-based classification methods are becoming more common outside of aurora image classification. Similar to aurora classification, there is a study that used CNN for cloud shape prediction [21]. Another study similar to our research reported the use of a CNN model for facial diagnosis [22]. In their study, they proposed an approach to apply a CNN that has performed face recognition to face diagnostics by transfer learning. One of the reasons why deep learning methods are now widely used is the high generalization performance of deep learning. It has been argued that the SGD (stochastic gradient descent) method used in parameter optimization is largely responsible for the generalization performance of DNN [23]. Furthermore, research on techniques to improve generalization performance is also ongoing [24]. In our research, we have also introduced approaches to improve generalization performance. For example, we have introduced early stopping to suppress overfitting and data augmentation to improve generalization performance.

### 2.2. Deep Metric Learning

Metric learning is a method of training machine learning models based on the distances between features in the data. In recent years, there has been a remarkable development of new metric learning methods that combine metric learning and deep learning. This method is called deep metric learning. Deep metric learning [25] was proposed by Hoffer et al. in 2014. Specifically, they proposed the Triplet Network and calculated distance in the process of loss calculation of the network. The Triplet Network uses three inputs. First, it selects the anchor data and then selects one sample that has the same label as the anchor label and one sample that has a different label. The three data are then inputted into three neural networks that share the structure and parameters. Then, they calculated the L2 norm for these three outputs and trained the network using a loss function, mean squared error. In 2015, Schroff et al. published a paper on face identification using deep metric learning [5]. In their study, they combined three networks used for deep metric learning into one. To achieve this, they made a sampling algorithm to choose three samples in a mini-batch, similar to the Triplet Network. In their study, it was important to know how to select the three samples. They set up a margin for the distance from the anchor sample in order to make the learning process more efficient, and selected samples that were suitable for learning based on the margin as follows:

(1) $$d_{ap}\leq d_{an}\leq d_{ap}+m$$

$d_{ap}$ represents a distance between an anchor sample and a positive sample, $d_{an}$ represents a distance between an anchor sample and a negative sample, and *m* represents a margin. The distance between the negative sample is farther than the positive sample and the anchor (not hard negative) and closer than the distance between the margin and the positive sample (not easy negative) by Equation (1). It prevents the system from falling into local solutions during learning, and at the same time suppresses the effects of outliers. Using these distances, triplet loss (Triplet Margin Loss) is defined as follows.

(2) $$L_{ap}={\left[d_{ap}-d_{an}+m\right]}_{+}$$

where *m* represents the margin for the sampling method. In deep metric learning, two aspects are important: the design of the loss function and the design of the triplet selection. These improvement methods were proposed one after another. In 2016, Sohn et al. proposed *N* pair multi-class loss [26], where triplet loss had a single negative sample for the anchor, while *N* pair multi-class loss samples from each of the N−1 classes to which the anchor does not belong. In 2019, a loss function that considers both intra-pair and extra-pair similarity, multi-similarity loss [27], was proposed. However, these metric learning methods are difficult to handle because they rely heavily on the accuracy of the triplet selection. Therefore, a method that does not use sampling was developed. This method is SphereFace [28]. Furthermore, CosFace [29] and ArcFace [30] have been developed as improved versions of SphereFace. In this study, in addition to deep metric learning using Triplet Margin Loss, we also trained the network using Additive Angular Margin Loss (ArcFace) [30]. The Additive Angular Margin Loss is expressed by Equation (3).

(3) $$L=-\frac{1}{m}\sum_{i=1}^{m}\log\frac{e^{s(\cos\theta_{yi}+m)}}{e^{s(\cos\theta_{yi}+m)}+\sum_{j=1,j\neq y_{i}}^{n}e^{s\cos\theta_{j}}}$$

where *m* represents margin and *s* represents scale. By setting the margin *m*, the network can be trained so that data of the same class are placed closer together in the feature space and data of different classes are placed farther apart.

Based on previous studies of aurora image classification using deep learning, we considered it necessary to take into account the fine features of aurora images for this task. Deep metric learning is a technique that is good at learning fine-grained features, as can be seen from the fact that it has developed mainly from studies of face identification. We hypothesized that deep metric learning methods could contribute to learning fine features, which have been considered difficult in conventional aurora image identification. We will describe the model in more detail in the next section.

## 3. Proposed Method

**Figure 1:**
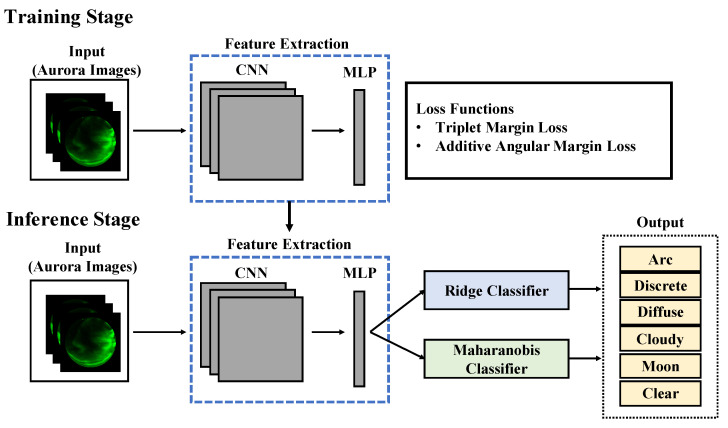
Inference framework for aurora image classification.

In this study, we propose a classification method using deep metric learning. Figure 1 shows the architecture of our classification model. First, we describe the training method for the classification model. The model is composed of a CNN, which serves as the backbone for embedding images, and a Multi-Layer Perceptron (MLP), which further reduces features to arbitrary dimensions. We used ResNet, whose accuracy has been noted in previous studies [13,14], for our CNN. The MLP is a neural network composed of three linear layers and MLP compressed the feature vector to 16 dimensions. We trained this model with Triplet Margin Loss or Additive Angular Margin Loss (ArcFace).

Next, we describe the inference methods. We fixed the parameters of the pre-trained model. Then, we used it as a feature extractor to convert images into feature vectors. We classified the feature vectors into six classes using two classification methods. The first classification method is ridge regression. Ridge regression is one of the linear regression models, where the L2 norm is introduced as a regularization term in linear regression to prevent overfitting. We trained it using the features from the training images and predicted the class of the test images. The second classification method is based on the Mahalanobis distance. Mahalanobis distance is a measure between a sample point and a distribution. The Mahalanobis distance *d* from a vector *y* to a distribution with mean μ and covariance Σ can be expressed by Equation (4).

(4) $$d=\sqrt{{\left(y-\mu \right)}^{T}\Sigma^{-1}\left(y-\mu \right)}$$

where *T* represents transpose. This distance indicates how far *y* is from the mean in standard deviation units. Using this distance, we were able to determine which cluster the test data belonged to.

## 4. Experiments

### 4.1. Data Splitting and Evaluation Method

**Figure 2:**
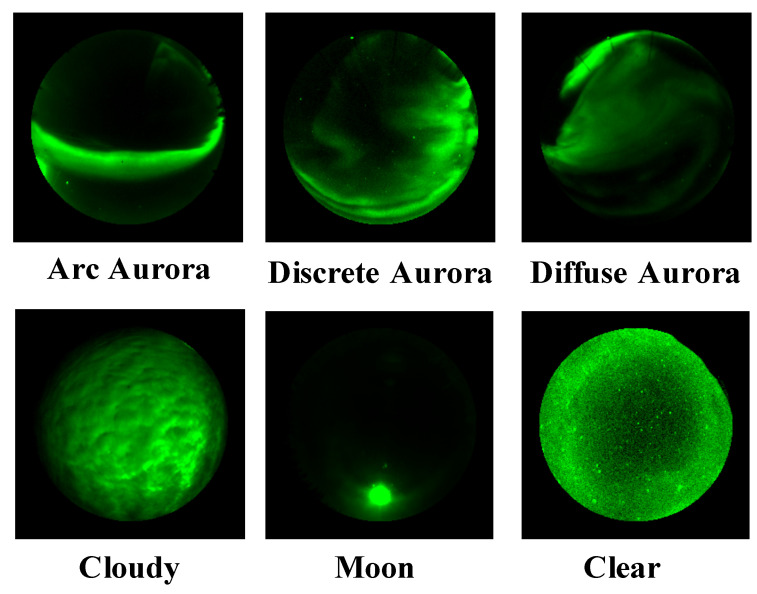
Sample images of TEMIS Ground-Based All-Sky Imager (ASI) dataset.

We used the THEMIS Ground-Based All-Sky Imager (ASI) dataset used in the study of Clausen [6]. This dataset consists of 5824 labeled images. It has six labels: Arc, Diffuse, Discrete, Cloudy, Moon, and No Aurora. Figure 2 shows sample images of the THEMIS Ground-Based ASI dataset. Since the number of images per label was different, we aligned the number of images per label for our experiments. We randomly selected 600 images from each of the six classes and used 3600 images as training data. Using the same selection method, 600 images were selected from the dataset for test images to evaluate the performance of our model. A common problem in training machine learning models is that the model overfits the training image. Therefore, we prepared validation images to check the robustness of the model. Table 1 shows the number of images of each phase. The images for this validation were selected from the training images. For each category, 100 images were selected, for a total of 600 images to evaluate the performance of the model. This process is called cross-validation, and we did it four times. In order to achieve this validation, we used a stratified-fold of scikit-learn [31].

To demonstrate the usefulness of our proposed method, we conducted two more comparative experiments. The first is a previous study using inceptionV4 and ridge regression [6]. For the second comparison experiment, we used a model fine-tuned with aurora image data against a CNN pre-trained on ImageNet. This method has been used frequently in recent studies [13,19,20] on aurora image classification. In addition, the effect of metric learning is important in this research. Therefore, we conducted experiments without metric learning as comparative ablation experiments.

### 4.2. Details of Model and Model Training Methods

We created our deep metric learning models using pytorch-metric-learning [32], which is a library of deep metric learning. We used ResNet as the backbone of our classification model and experimented with two patterns, ResNet18 and ResNet50. In particular, we use ResNet18 and ResNet50, which are pre-trained with ImageNet. The usefulness of models pre-trained with ImageNet has been shown in a previous study [13]. We used ResNet because previous studies [13,19] have reported that VGG, AlexNet, and ResNet are commonly used, with ResNet being the most accurate CNN among them. In addition, we trained our CNN model with two different deep metric learning methods. The parameters for each method are listed in Table 2. We adjusted these parameters for each method. To evaluate the performance of our model during training, we used the k-nearest neighbor method and implemented early stopping, which terminates the training based on the score. We used a single NVIDIA Tesla P100 GPU for this experiment.

In the inference stage, we used ridge regression and Mahalanobis distance for the classification. We used the ridge regression classifier implemented in sckit-learn [31]. Therefore, the ridge regression classifier is trained in a one-versus-all approach.

### 4.3. Data Preprocessing

We are here to discuss image preprocessing. First, all images were resized to 256 × 256. Furthermore, we introduced horizontal and vertical flipping and rotation, and random transformation of image brightness and contrast during training. We used albumentations, a python library for image augmentation.

## 5. Results

**Figure 3:**
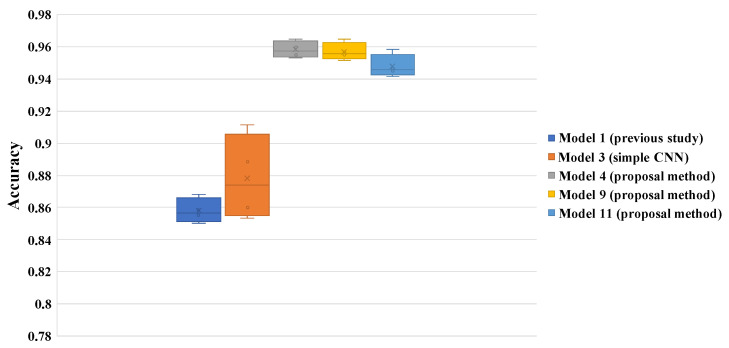
Results of aurora image classification.

The results of our experiments are shown in Table 3 and Figure 3. Table 3 shows accuracy scores of test images. The highest average score is 0.95833 using deep metric learning and Mahalanobis distance (Model 4). This score is about 8% higher than the comparative method using ResNet18 (Model 2). It is also about 10% higher than the model of the previous study using Inception-v4 (Model 1). Furthermore, Figure 3 shows that models using deep metric learning have less variation in results from fold to fold. Table 4 and Table 5 show the confusion matrix of Model 1 and Model 4, respectively. The results in these tables show that the use of deep metric learning improves the correct response rate for all labels. We can also confirm that the arc shape was the most difficult to discriminate among the six labels.

**Figure 4:**
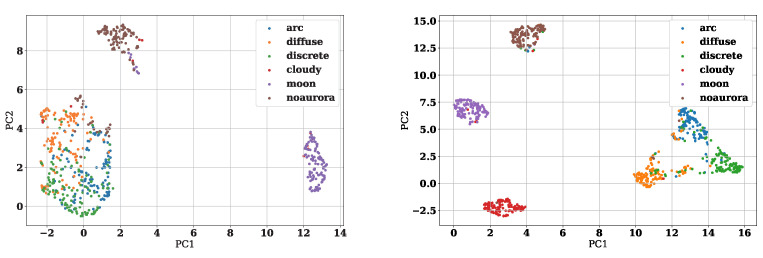
These are features obtained by the Triplet Margin Loss model compressed to 2 dimensions. The left part shows the features of the first epoch of fold 0 and the lower part is one of epoch 15 of fold 0.

**Figure 5:**
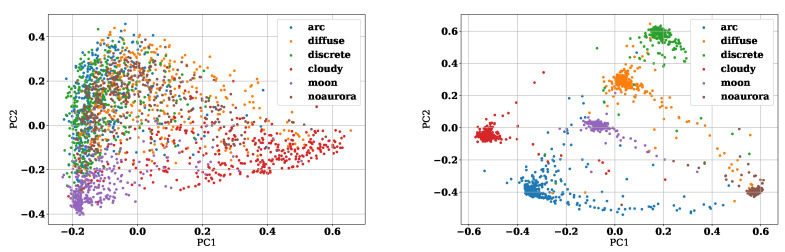
These are features obtained by the ArcFace model compressed to 2 dimensions. The left part shows the features of the first epoch of fold 0 and the lower part is one of epoch 45 of fold 0.

**Figure 6:**
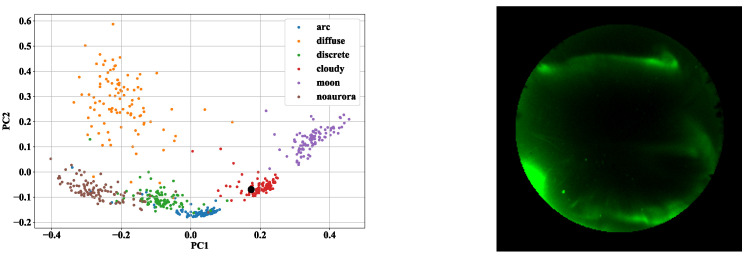
Analysis results of all training data. This shows an image that is identified as cloudy on the cluster but is actually the moon. The large black dot on the graph indicates the embedding to which this image corresponds.

Next, we visualize feature vectors encoded from images to see whether deep metric learning is effective. We compressed these features into two dimensions using principal component analysis (PCA) and plotted the first principal component on the *x*-axis and the second principal component on the *y*-axis. Figure 4 and Figure 5 show the results. Figure 4 illustrates the feature vectors obtained by the Triplet Margin Loss model (Models 4 and 5), and Figure 5 illustrates the feature vectors obtained by the ArcFace model (Models 8 and 9). The left figure of Figure 5 shows features of the training data during the training of the first epoch of fold 0. On the other hand, the right figure shows the feature space of the training data at 15 epochs of fold 0. From Figure 4, we can see that clusters can be clearly separated for each label in the feature space as deep metric learning progresses. It also can be seen that the clusters of arc aurora, diffuse aurora, and discrete aurora are in close proximity to each other in the feature space of epoch 15. It indicates that these three auroras have similar characteristics and are more difficult to classify than the other categories. Although Triplet Margin Loss and ArcFace are different deep metric learning methods, it is clear that both are able to generate excellent feature spaces. The advantage of visualizing feature space is not only to get a rough idea of the characteristics of the data but also to check the data one by one, which is difficult for the model to infer correctly. Figure 6 shows the feature space of all training data. The large black dot in Figure 6 indicates the location of the image that is closest to the cloudy aurora cluster but is actually the moon aurora. This aurora image is attached at the right of Figure 6. Thus, by analyzing images that are difficult for the model to interpret, we can make great use of them to improve the model in the future.

## 6. Discussion

These results show that deep metric learning can significantly improve the accuracy of classifying aurora images. In addition, we think that the feature vectors of deep metric learning have the advantage of visualizing images that are difficult to classify. There are two possible ways to improve a classification model in the future: the first is to optimize the hyperparameters. The optimal hyperparameter value varies depending on the training method. As shown in Table 2, the appropriate parameters for the two deep metric learning methods also differed significantly. We think that more rigorous tuning of these parameters will improve accuracy. The second is the design of the feature extractor. In this experiment, we used ResNet18 and ResNet50. There was no significant difference between these two backbones. Currently, powerful deep learning models such as EfficientNet [33] and vision transformer [34] are being used in the image recognition field. The features of aurora images are smaller than typical images such as ImageNet. Therefore, we cannot say in general that it is useful to use deep neural nets that require many parameters as a backbone, but we think it should be considered.

In addition, we compared the cost of the proposed model using deep metric learning (ArcFace) with a conventional model using Cross Entropy Loss. We measured the training time for 10 epochs, using 3600 training images as 1 epoch. The conventional CNN took 12.27 s to train one epoch on average. On the other hand, even when ArcFace is used, it only took 16.03 s to train the model. Moreover, we were able to infer 600 images in 17.67 s. We used a single NVIDIA Tesla P100 GPU for this experiment. These results show that, to begin with, model training for aurora image classification can be performed at a low cost, and that the cost does not change significantly even when deep metric learning is used.

In our study, we used the THEMIS Ground-Based All-Sky Imager (ASI) dataset [19]. However, the Yellow River Station (YRS) dataset is also a major dataset as seen in the paper [13]. One of the differences between YRS and ASI is that ASI is a color image dataset, while YLR is a black and white image dataset. We conducted additional experiments by converting ASI to grayscale considering the applicability of the proposed method to YRS. In this case, we measured the accuracy when the ASI dataset was black and white images as a simple verification. Table 6 shows the results. As shown in Table 6, although some accuracy degradation was observed, it was found that it was possible to classify with higher accuracy than finetuning CNN. Based on this experimental result, it is expected that this method is also effective for YRS datasets containing black and white images. In the future, we would like to conduct experiments utilizing the YRS dataset based on these results.

## 7. Conclusions

We used deep metric learning to classify aurora images. The experimental results show that deep metric learning can dramatically improve the recognition accuracy of aurora images. We hypothesized that metric learning would be effective for aurora image classification by considering the similarities between the face recognition task and the aurora image classification task. The results of our experiments demonstrate that our hypothesis has been proven.

In addition, deep metric learning allows for visual confirmation of the features in the training data. This allowed us to identify samples that the classifier had difficulty with, which is very useful for improving accuracy in future analyses. Moreover, different hyper-parameter settings are also important to evaluate the validity of the proposed method. We would like to investigate the robustness of the proposed method in the future.

## Figures and Tables

**Table 1:** The number of images in each phase.

Phase	A Number of Images
Training	3000 (500 images × 6 classes)
Validation	600 (100 images × 6 classes)
Evaluation	600 (100 images × 6 classes)

**Table 2:** Parameters of deep metric learning.

Parameters	Triplet Margin Loss	ArcFace
Epoch	40	50
Learning rate	0.001	0.01
Dimensions of feature vectors	16	256
Batch size	32	64
Optimizer	Adam	SGD

**Table 3:** Classification results for the six classifications. Model 1 through Model 3 are prior studies, and Model 4 through Model 11 are proposed methods using deep metric learning. The details column provides details of the model. “Backbone” describes the main CNN model, and “Classifier” describes the classification method. In particular, Model 2 and Model 3 are described as “End to End" because feature extraction and classification are performed in one step.

Model	Details		Fold1	Fold2	Fold3	Fold4	Average
Model 1	Backbone	InceptionV4	0.85000	0.86833	0.85500	0.85833	0.85792 (SD 0.00670)
	Classifier	Ridge Regression					
Model 2	Loss Function	Cross Entropy Loss	0.84667	0.87333	0.88500	0.89333	0.87458 (SD 0.01761)
	End to End	ResNet18					
Model 3	Loss Function	Cross Entropy Loss	0.88833	0.91167	0.85333	0.86000	0.87833 (SD 0.02330)
	End to End	ResNet18					
Model 4	Loss Function	Triplet Margin Loss	0.95333	0.96000	0.95500	0.96500	**0.95833 (SD 0.00457)**
	Backbone	ResNet18					
	Classifier	Mahalanobis Distance					
Model 5	Loss Function	Triplet Margin Loss	0.95500	0.95333	0.95667	0.95000	0.95375 (SD 0.00247)
	Backbone	ResNet18					
	Classifier	Ridge Regression					
Model 6	Loss Function	Triplet Margin Loss	0.94500	0.94833	0.94500	0.93000	0.94208 (SD 0.00711)
	Backbone	ResNet50					
	Classifier	Mahalanobis Distance					
Model 7	Loss Function	Triplet Margin Loss	0.96167	0.95000	0.95000	0.94500	0.95167 (SD 0.00613)
	Backbone	ResNet50					
	Classifier	Ridge Regression					
Model 8	Loss Function	ArcFace	0.93333	0.95000	0.95500	0.95667	0.95250 (SD 0.00923)
	Backbone	ResNet18					
	Classifier	Mahalanobis Distance					
Model 9	Loss Function	ArcFace	0.95167	0.95667	0.95500	0.96500	**0.95583 (SD 0.00491)**
	Backbone	ResNet18					
	Classifier	Ridge Regression					
Model 10	Loss Function	ArcFace	0.94167	0.95333	0.95667	0.95167	0.95250 (SD 0.00559)
	Backbone	ResNet50					
	Classifier	Mahalanobis Distance					
Model 11	Loss Function	ArcFace	0.94667	0.94167	0.94500	0.95833	0.94583 (SD 0.00628)
	Backbone	ResNet50					
	Classifier	Ridge Regression					

**Table 4:** Confusion matrices in the case of classification into six classes (Model 1).

		Estimated Results					
		Arc	Diffuse	Discrete	Cloudy	Moon	No Aurora
**Class**	arc	83	6	9	0	1	2
	Diffuse	11	76	7	1	0	6
	Discrete	13	8	77	1	1	1
	Cloudy	1	3	2	93	2	1
	Moon	0	0	1	1	98	0
	Noaurora	4	2	5	0	1	89

**Table 5:** Confusion matrices in the case of classification into six classes (Model 4).

		Estimated Results					
		Arc	Diffuse	Discrete	Cloudy	Moon	No Aurora
**Class**	arc	89	3	3	0	0	0
	Diffuse	4	93	2	0	0	1
	Discrete	6	4	95	0	2	1
	Cloudy	0	0	0	100	0	0
	Moon	0	0	0	0	98	1
	Noaurora	1	0	0	0	0	97

**Table 6:** Image classification results on grayscaled ASI dataset using Model 6.

	Fold1	Fold2	Fold3	Fold4
Accuracy	0.92933	0.93200	0.92267	0.90267

## Data Availability

Not applicable.

## References

[1] Mikko S., Noora P. (2012). Numeric image features for detection of aurora. IEEE Geosci. Remote Sens. Lett..

[2] Qiuju Y., Jimin Z., Zejun H., Heng Z. (2012). Auroral sequence representation and classification using hidden Markov models. IEEE Trans. Geosci. Remote Sens..

[3] Andreas K., Kristoffer W., Derek M., Noora P. (2020). Auroral Image Classification With Deep Neural Networks. J. Geophys. Res. Space Phys..

[4] Kaiming H., Xiangyu Z., Shaoqing R., Jian S. Deep residual learning for image recognition. Proceedings of the IEEE Conference on Computer Vision and Pattern Recognition.

[5] Florian S., Dmitry K., James P. FaceNet: A Unified Embedding for Face Recognition and Clustering. Proceedings of the Conference on Computer Vision and Pattern Recognition (CVPR).

[6] Lasse B.N.C., Hannes N. (2015). Automatic classification of auroral images from the Oslo auroral THEMIS (OATH) data set using machine learning. J. Geophys. Res. Space Phys..

[7] Syrjäsuo M.T., Donovan E.F. (2004). Diurnal auroral occurrence statistics obtained via machine vision. Ann. Geophys..

[8] Syrjäsuo M.T., Donovan E.F. Using relevance feedback in retrieving auroral images. Proceedings of the 4th IASTED International Conference on Computational Intelligence.

[9] Han B., Qiu W. (2013). Aurora images classification via features salient coding. J. Xidian Univ..

[10] Han B., Yang C., Gao X. (2013). Aurora image classification based on LDA combining with saliency information. J. Softw..

[11] Xi Y., Xinbo G., Bin S., Dong Y. (2013). Aurora image search with contextual CNN feature. Neurocomputing.

[12] Xi Y., Xinbo G., Bin S., Nannan W., Dong Y. (2018). ASI aurora search: An attempt of intelligent image processing for circular fisheye lens. Opt. Express.

[13] Yanfei Z., Richen Y. (2020). Automatic Aurora Image Classification Framework Based on Deep Learning for Occurrence Distribution Analysis: A Case Study of All-Sky Image Data Sets From the Yellow River Station. J. Geophys. Res. Space Phys..

[14] Krizhevsky A., Sutskever I., Hinton G.E. (2017). Imagenet classification with deep convolutional neural networks. Commun. ACM.

[15] Simonyan K., Zisserman A. (2014). Very deep convolutional networks for large-scale image recognition. arXiv.

[16] Chuang N., Jun Z., Qian W., Jimin L. (2018). Weakly supervised semantic segmentation for joint key local structure localization and classification of aurora image. IEEE Geosci. Remote Sens. Lett..

[17] Qiuju Y., Penghui Z. (2020). Representation and Classification of Auroral Images Based on Convolutional Neural Network. IEEE Geosci. Remote Sens. Lett..

[18] Aoi U., Mitsuharu M. (2021). Extension of Image Data Using Generative Adversarial Networks and Application to Identification of Aurora. IEEE Geosci. Remote. Sens. Lett..

[19] Pascal S., Lasse B.N.C., Wojciech M., Hannes N. (2021). Transfer Learning Aurora Image Classification and Magnetic Disturbance Evaluation. J. Geophys. Res. Space Phys..

[20] Qiuju Y., Yingying W., Jie R. (2022). Auroral Image Classification With Very Limited Labeled Data Using Few-Shot Learning. IEEE Geosci. Remote Sens. Lett..

[21] Mengyang Z., Chorng H.C., Wenbin X., Zhou X., Jinyong H. (2020). Cloud shape classification system based on multi-channel cnn and improved fdm. IEEE Access.

[22] Bo J., Leandro C., Nuno G. (2020). Deep Facial Diagnosis: Deep Transfer Learning From Face Recognition to Facial Diagnosis. IEEE Access.

[23] Suriya G., Blake W., Srinadh B., Behnam N., Nathan S. (2017). Implicit Regularization in Matrix Factorization. arXiv.

[24] Qinghe Z., Mingqiang Y., Jiajie Y., Qingrui Z., Xinxin Z. (2018). Improvement of Generalization Ability of Deep CNN via Implicit Regularization in Two-Stage Training Process. IEEE Access.

[25] Elad H., Nir A. (2014). Deep metric learning using Triplet network. arXiv.

[26] Sohn K. (2016). Improved deep metric learning with multi-class N-pair loss objective. Adv. Neural Inf. Process. Syst..

[27] Wang X., Han X., Huang W., Dong D., Scott M.R. Multi-similarity loss with general pair weighting for deep metric learning. Proceedings of the IEEE/CVF Conference on Computer Vision and Pattern Recognition.

[28] Liu W., Wen Y., Yu Z., Li M., Raj B., Song L. Sphereface: Deep hypersphere embedding for face recognition. Proceedings of the IEEE Conference on Computer Vision and Pattern Recognition.

[29] Wang H., Wang Y., Zhou Z., Ji X., Gong D., Zhou J., Li Z., Liu W. Cosface: Large margin cosine loss for deep face recognition. Proceedings of the IEEE Conference on Computer Vision and Pattern Recognition.

[30] Jiankang D., Jia G., Niannan X., Stefanos Z. (2018). ArcFace: Additive Angular Margin Loss for Deep Face Recognition. arXiv.

[31] Scikit-Learn. https://scikit-learn.org/stable/.

[32] Pytorch-Metric-Learning. https://github.com/KevinMusgrave/pytorch-metric-learning.

[33] Tan M., Le Q. Efficientnet: Rethinking model scaling for convolutional neural networks. Proceedings of the International Conference on Machine Learning.

[34] Alexey D., Lucas B., Alexander K., Dirk W., Xiaohua Z., Thomas U., Mostafa D., Matthias M., Georg H., Sylvain G. (2020). An Image is Worth 16x16 Words: Transformers for Image Recognition at Scale. arXiv.

